# Gametocyte carriage of *Plasmodium falciparum* (*pfs25*) and *Plasmodium vivax* (*pvs25*) during mass screening and treatment in West Timor, Indonesia: a longitudinal prospective study

**DOI:** 10.1186/s12936-021-03709-y

**Published:** 2021-04-09

**Authors:** Ayleen Kosasih, Cristian Koepfli, M. Sopiyudin Dahlan, William A. Hawley, J. Kevin Baird, Ivo Mueller, Neil F. Lobo, Inge Sutanto

**Affiliations:** 1 PhD Programme in Biomedical Sciences, Medical Faculty, Universitas Indonesia, Jakarta, Indonesia; 2 Eijkman-Oxford Clinical Research Unit, Jakarta, Indonesia; 3 Indonesian Medical Education and Research Institute, Jakarta, Indonesia; 4 Eck Institute for Global Health, University of Notre Dame, Notre Dame, IN USA; 5 PT Epidemiologi Indonesia, Jakarta, Indonesia; 6 UNICEF, Jakarta, Indonesia; 7 Center for Tropical Medicine and Global Health, Nuffield Department of Medicine, University of Oxford, Oxford, UK; 8 Infection & Immunity Division, Walter & Eliza Hall Institute, Melbourne, Australia; 9 Department of Parasitology, Medical Faculty, Universitas Indonesia, Jakarta, Indonesia

**Keywords:** Gametocyte, *Pfs25*, *Pvs25*, Mass screening and treatment

## Abstract

**Background:**

A goal of malaria epidemiological interventions is the detection and treatment of parasite reservoirs in endemic areas—an activity that is expected to reduce local transmission. Since the gametocyte is the only transmissible stage from human host to mosquito vector, this study evaluated the pre and post presence of gametocytes during a mass screening and treatment (MST) intervention conducted during 2013 in East Nusa Tenggara, Indonesia.

**Methods:**

RT-qPCR targeting *pfs25* and *pvs25* transcripts—gametocyte molecular markers for *Plasmodium falciparum* and *Plasmodium vivax*, respectively, was performed to detect and quantify gametocytes in blood samples of *P. falciparum* and *P. vivax-*infected subjects over the course of the MST study. The presence of both asexual and sexual parasites in microscopic and submicroscopic infections was compared from the start and end of the MST, using proportion tests as well as parametric and non-parametric tests.

**Results:**

Parasite prevalence remained unchanged for *P. falciparum* (6% = 52/811 *versus* 7% = 50/740, p = 0.838), and decreased slightly for *P. vivax* (24% = 192/811 *versus* 19% = 142/740, p = 0.035) between the MST baseline and endpoint. No significant difference was observed in gametocyte prevalence for either *P. falciparum* (2% = 19/803 *versus* 3% = 23/729, p = 0.353, OR = 1.34, 95%CI = 0.69–2.63), or *P. vivax* (7% = 49/744 v*ersus* 5% = 39/704, p = 0.442, OR = 0.83, 95%CI = 0.52–1.31). Even though there was an insignificant difference between the two time points, the majority of parasite positive subjects at the endpoint had been negative at baseline (*P. falciparum*: 66% = 29/44, *P. vivax*: 60% = 80/134). This was similarly demonstrated for the transmissible stage—where the majority of gametocyte positive subjects at the endpoint were negative at baseline (*P. falciparum*: 95% = 20/21, *P. vivax*: 94% = 30/32). These results were independent of treatment provided during MST activities. No difference was demonstrated in parasite and gametocyte density between both time points either in *P. falciparum* or *P. vivax*.

**Conclusion:**

In this study area, similar prevalence rates of *P. falciparum* and *P. vivax* parasites and gametocytes before and after MST, although in different individuals, points to a negligible impact on the parasite reservoir. Treatment administration based on parasite positivity as implemented in the MST should be reevaluated for the elimination strategy in the community.

*Trial registration* Clinical trials registration NCT01878357. Registered 14 June 2013, https://www.clinicaltrials.gov/ct2/show/NCT01878357.

**Supplementary Information:**

The online version contains supplementary material available at 10.1186/s12936-021-03709-y.

## Background

The sexual stage of malaria-causing parasites—gametocytes, originate from the asexual cycle in human red blood cells. However, among *Plasmodium* infecting reptiles and birds, gametocytes may also be produced from secondary exo-erythrocytic schizogony [1]. In the blood, only a small proportion of the asexual parasites differentiate into gametocytes [2]. In addition, gametocytes do not replicate in the blood, so their quantity depend on the number of progenitor asexual stages in the bloodstream [3]. There are characteristic differences in gametocyte development and longevity between the dominant malaria species [2]. In *Plasmodium falciparum*, the development of gametocytes typically takes 8–10 days where they sequester largely in the bone marrow until maturation [2]. Mature gametocytes re-enter the circulatory system where they remain for 3–4 weeks and are spontaneously cleared if not ingested by a mosquito vector [4]. Conversely, *Plasmodium vivax* gametocyte development takes 48 h to maturity with all stages of gametocytes present in the peripheral blood at the same time and are cleared within three days post maturation [2, 5]. It is not known if that clearance represents sequestration to another compartment, e.g. the microvasculature of skin accessible to anopheline mosquitoes.

Human-to-mosquito transmission requires the uptake of male and female gametocytes [2]. The infectivity of this stage to mosquitoes is positively associated with gametocyte density [6–8] with variations seen between species and study areas [9–11]. Non-linear relationships between gametocyte density and mosquito infection rates have been reported in *P. falciparum* [2], whereas in *P. vivax*, the relationship was demonstrated to be sigmoidal [8]. Furthermore, the gametocyte sex ratio, multiplicity of infection (MOI) [2], species, specific clones [12], and mixed infections [13] were also reported to impact transmission events. In addition, host factors also may affect gametocyte infectiousness [14], e.g. gametocytes from children were reported to be more infectious than those from adults, partly due to the higher gametocyte density in the younger age group [15]. Host immunity has also been documented to inhibit oocyst development in the mosquito [2, 16]. After about 10–12 days, oocysts develop into sporoblasts, which contains sporozoites [17]. Sporozoite will egress the sporoblast membrane and migrate to the salivary gland, ready to be transmitted [17].

The human host, when infected with *Plasmodium*, may become symptomatic or asymptomatic [18]. Symptomatic infections can usually be detected microscopically, while asymptomatic infections are frequently below the detection threshold of microscopic examination and require more sensitive molecular detection methodologies [18]. These submicroscopic infections are reportedly less infectious than those detectable by microscopy, however, they may contribute more to transmission due to a much higher frequency in the population [6, 7]. Symptomatic individuals usually have a higher treatment-seeking rates and, on diagnosis, are treated with both a gametocytocidal and a blood schizonticide agent, such as primaquine and artemisinin-based combination therapy (ACT). In addition, the administration of ACT has been demonstrated to have a temporary prophylactic effect against new infections of blood [19–21].

Surveillance in the context of elimination strategies seeks to identify and treat asymptomatic carriers of malaria [22]. A consequent reduction in the human parasite reservoir is hence expected to lower the transmission rate [22]. Mass drug administration (MDA) and mass screen and treat (MST) strategies have been developed and studied as intervention strategies [23–26]. Most MST studies have reported the prevalence and/or incidence of parasitaemia as the outcomes, however data on the infective gametocytes has not been described despite it having an important role in transmission [27–29]. This study analysed the gametocyte carrier status of population receiving MST intervention [26] by examining gametocyte specific transcripts, *pfs25* and *pvs25*.

## Methods

### Study area

This study was conducted in Wewiku subdistrict, Belu district, East Nusa Tenggara province, in eastern Indonesia (June–September 2013). The study area was reported to have the highest malaria endemicity in the province. The annual parasite incidence (API) per 1000 population was 72 and 124 in 2011 and 2012, respectively (Belu district health office, personal communication), whereas in the entire East Nusa Tenggara province it was 25.8 and 21.1 during the same period [30]. Malaria incidence peaked during the months of August and September which coincided with the end of this study [26]. *Anopheles barbirostris* was reported as the major malaria vector [26]. Other less dominant vectors included *Anopheles subpictus* and *Anopheles vagus* [26]. A more detailed description on the study site has previously been reported [26].

### Sample collection

The original cluster-randomized trial evaluated the impact of MST conducted (a) twice, and (b) three times, both within 3 months [26]. Samples were collected in June (round-1), July (round-2) and August (round-3) [26]. Positivity for malaria parasites by microscopy was 8% in both groups at baseline, and 9% each at the last round of MST [26]. MST exerted no impact on measured incidence of diagnosed infections in both groups (RR = 0.89, 95%CI 0.43–1.83) [26]. Therefore, in this study, round-1 of MST from both treatment groups were pooled and considered the ‘baseline’, while the last time-point (round-3) from both groups was also pooled and considered the ‘endpoint’.

Finger-pricked blood samples from each of the study subjects were collected for malaria diagnosis using microscopy and 250 µL were also collected in EDTA tubes. Blood slides were air-dried overnight prior to Giemsa staining. Upon staining, microscopic readings were performed on the same day. *P. falciparum* and *P. vivax*-positive blood samples were screened for glucose-6-phosphate dehydrogenase (G6PD) deficiency using a fluorescence spot test (Trinity Biotech, Ireland), and subjects with deficient or intermediate G6PD activities were not given primaquine. The time required from the sample collection until treatment administration were 2–3 days. All microscopy-positive subjects were treated with dihydroartemisinin–piperaquine (DHP) and primaquine. Treatment consisted of DHP (each tab contains 40 mg dihydroartemisinin and 320 mg piperaquine) for 3-day with daily doses as follows: ≥ 60 kg = 4, 41–59 kg = 3, 31–40 kg = 2, 18–30 kg = 1.5, 11–17 kg = 1, 6–10 kg = 0.5 and ≤ 5 kg = 0.25 tablet(s). Primaquine administration for *P. falciparum* were 0.75 mg/kgBW single dose: ≥ 60 kg = 3, 41–59 kg = 2, 31–40 kg = 2, 18–30 kg = 1.5, 11–17 kg = 0.75 tablet(s). For *P. vivax*, the daily dose for 14 days were 0.25 mg/kgBW: 1, 1, 0.75, 0.5, 0.25 tablet for the same weight group. A pill cutter was used to cut 15 mg of primaquine tablet to meet the required dose according to the body weight. A pill grinder was used to make powder with added saccharine for small children, e.g. < 6 year old, who were unable to swallow tablets. Quality control with cross-checking of microscopic reading was performed post-hoc with kappa value = 0.82 and 0.85 for *P. falciparum* and *P. vivax*, respectively. Discordant speciation between microscopy and PCR was not an issue as same drugs were given for both species.

For RNA extractions, 50 µL of blood was mixed with 250 µL of RNAProtect (Qiagen, Germany) within 4 h of collection and stored at − 80 °C until further processing. During five years of storage, temperature was checked daily.

### Molecular analysis

**Fig. 1:**
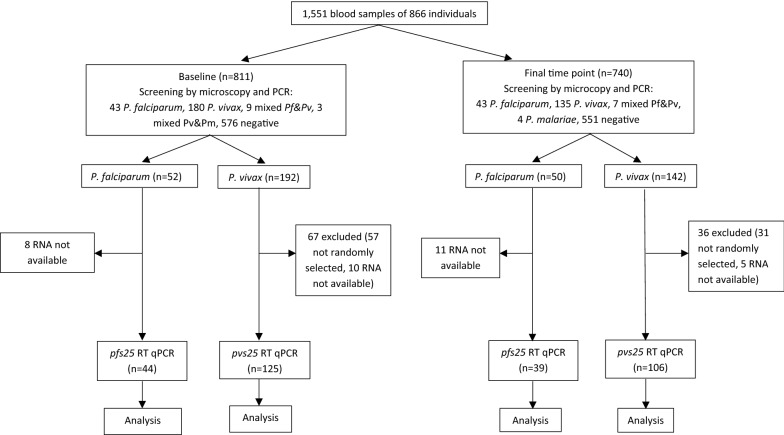
Study framework

Microscopic and PCR screening results have been reported in the parent study [26]. All *P. falciparum* and a set of randomly selected *P. vivax* positive samples underwent RNA extraction (Fig. 1) using the Quick RNA Mini-prep kit (Zymo Research, USA) according to manufacturer’s instruction. DNase treatment (included in the kit) was done during the process. *P. falciparum* and *P. vivax* gametocytes were quantified by two-step RT-qPCR of the gametocyte markers *pfs25* and *pvs25* [31]. Transcriptor First Strand cDNA kit (Roche) was used to generate cDNAs for each sample in triplicates. The presence of mRNA transcripts was verified using RT-qPCR targeting 18S rRNA [32], and only those positive for 18S were analysed for *pfs25* and *pvs25*. The *pfs25* and *pvs25* qPCR was conducted using FastStart Essential DNA SYBR Green master (Roche) with previously published primers [29]. Details on the assays are provided in the Additional file 1. Melting temperature (T_M_) for *P. falciparum* was 74–75 °C, and 79–80 °C for *P. vivax* (Additional file 2). For quantification, series of plasmid harbouring target sequence with concentration of 10^5^, 10^4^, 10^3^, 10^2^, 10, 5, and 1 copy(ies) per reaction were run in triplicate and a standard curve was generated for each run. Negative (no template) controls were included in triplicates. Minus-reverse transcriptase (−RT) qPCR using *pfs25* and *pvs25* was performed on 23% (19/83) of randomly selected of *P. falciparum* and 17% (40/231) of *P. vivax* RNA samples to check for genomic DNA contamination. Sixteen of 19 *P. falciparum* demonstrated no contamination with DNA while three showed the presence of DNA—with 0.01–1.28% of *pfs25* RT qPCR result. Thirty-six of 40 *P. vivax* demonstrated the absence of DNA, while four showed presence of 0.4%–2.5% DNA with *pvs25* RT qPCR results. The limit of detection (LOD) was assessed by running a serial dilution of the plasmid in quintuples and determined as 10 copies/µL for both *pfs25* and *pvs25*. Performance of the assay is described in Additional file 3. All triplicates of the cDNA were run and recorded as positive if a minimum of two of the three demonstrated a positive result. Quantities of the transcript were reported as the average transcript numbers of the replicates to the nearest CT values. Conversion of transcript numbers into gametocyte density was made by extrapolating transcript numbers to the standard curve generated from transcript numbers of the known gametocyte density in the microscopy-positive subjects. Regression coefficient (r) was 0.758 and 0.835 for *P. falciparum* and *P. vivax*, respectively. The trendline equation for *P. falciparum* was: 10^0.251 [−0.607–1.109]^ * (*pfs25* transcript numbers/µL)^0.621 [0.357–0.886]^, whereas for *P. vivax* was: 10^0.673 [0.198–1.149]^ * (*pvs25* transcript numbers/µL)^0.406 [0.264–0.548]^ (Additional file 4). Using this equation, one gametocyte corresponds roughly to 0.4 (0.1–50.2) *pfs25* transcripts/µL for *P. falciparum* and 0.02 (0.01–0.18) *pvs25* transcripts/µL for *P. vivax*. DNA from samples which were tested for gametocytes were quantified to assess the relationship between asexual and sexual stages. This was performed by species-specific 18S qPCR as described previously with some modification [33]. Details of the procedure and performance of the qPCR were shown in Additional files 1 and 3.

All laboratory analyses were performed blindly to subject identification (ID). Upon completion of all laboratory work, the results were linked to the subject ID, MST time point, and treatment status. All laboratory analyses were conducted at the Indonesian Medical Education and Research Institute (IMERI), Faculty of Medicine, University of Indonesia, Jakarta.

### Data analysis

This study was unable to be powered to detect any change in gametocyte prevalence during MST as originally planned given the much lower prevalence than estimation in the protocol. Moreover, the design of the study was modified to pre-post instead of a between-group analysis. Categorical variables were analysed using chi square test. Numerical variables were analysed using Student’s t-test when the distribution was normal or Mann–Whitney when it was not normal. 18S rRNA gene copies and *pfs25*/*pvs25* transcripts were log transformed for parametric statistical analyses. Linear regression was conducted to investigate the correlation between parasite density and *pfs25*/*pvs25* numbers. A p-value < 0.05 was considered as statistically significant. All analyses were performed using SPSS version 23 (IBM, Armonk, New York).

## Results

### Characteristic of the study subjects

A total 866 subjects participated in this study: 811 samples were collected at baseline and 740 at the endpoint. These subjects represented 78% of the total parent study (reference) samples. Age and gender of study subjects were comparable between baseline and endpoint (33% were ≤ 15 year of age and 47% were male). Furthermore, 685 (84%) subjects were sampled at both time points.

### *pfs25/pvs25* RT qPCR assay

Of the 102 *P. falciparum* positive samples by microscopy and/or PCR from the two time points, RNA extractions were not conducted in three samples due to insufficient blood volume, and 13 samples due to human error. Of the remaining 86 RNA samples, 3 were negative for 18S transcripts, with 83 remaining for *pfs25* analysis (Fig. 1). Of the 334 *P. vivax* positive samples by microscopy and/or PCR from the two time points, 246 were randomly selected for RNA extraction. Of these 246 RNA samples, 15 were negative for 18S transcripts—thus 231 samples were available for *pvs25* analysis (Fig. 1).

**Fig. 2:**
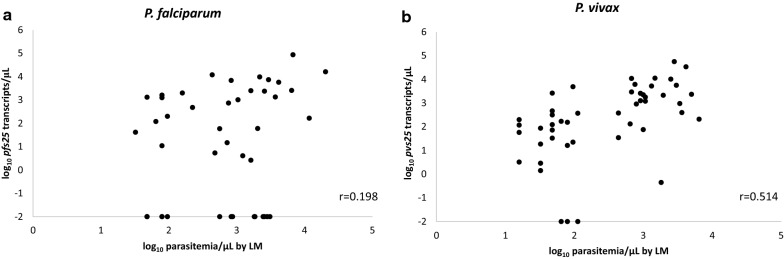
Correlation between gametocyte transcript numbers and parasitaemia density by light microscopy (LM) in *P. falciparum* (**a**) and *P. vivax* (**b**). In *P. falciparum*, no correlation was found between gametocyte transcript numbers and parasitaemia density by microscopy (r = 0.198, p = 0.202), whereas in *P. vivax*, positive correlation was demonstrated (r = 0.514, p < 0.001)

**Fig. 3:**
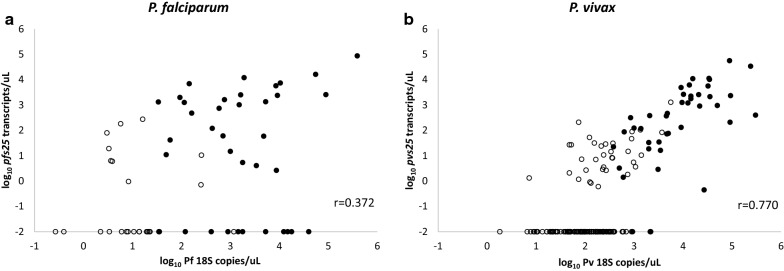
Correlation between gametocyte transcript numbers and 18S copies by qPCR in *P. falciparum* (**a**) and *P. vivax* (**b**). A positive correlation was demonstrated between 18S gene copy numbers/µL and gametocyte transcript numbers/µL for **a** *P. falciparum* (*pfs25*) as well as for **b** *P. vivax/pvs25*. Linear regression analyses both demonstrated a significance correlation (p < 0.001). Microscopic infections (●) were showing higher transcripts than submicroscopic infections (○)

In *P. falciparum*, gametocyte transcript numbers were not correlated with parasitaemia density by microscopy (r = 0.198, p = 0.202, Fig. 2a), whereas in *P. vivax*, positive correlation was demonstrated (r = 0.514, p < 0.001, Fig. 2b). However, gametocyte transcript numbers were positively correlated with 18S gene copy numbers for both species, with a stronger correlation observed for *P. vivax* than *P. falciparum* (*P. falciparum*: r = 0.372, p = 0.003; *P. vivax*: r = 0.770, p < 0.001, Figs. 3a, b).

### Prevalence and density of the parasite and gametocyte between baseline and endpoint

#### Plasmodium falciparum

Between baseline and endpoint, no significant difference in parasite prevalence was seen by microscopy, PCR, or either test (microscopy: 3% = 22/811 versus 5% = 34/740, p = 0.056, PCR: 6% = 49/811 versus 6% = 42/740, p = 0.829, either test: 6% = 52/811 versus 7% = 50/740, p = 0.838) (Table 1). No significant difference was also shown in parasite density by both microscopy and qPCR (median parasitaemia: 440 versus 860, p = 0.351; median 18S gene copies: 59.4 versus 713.5, p = 0.095) (Table 1). Furthermore, no significant difference was detected for gametocyte prevalence by microscopy, *pfs25*, or either test (microscopy: 1.4% = 11/811 versus 2% = 15/740, p = 0.328; *pfs25*: 2% = 19/803 versus 3% = 23/729, p = 0.353; either test: 2% = 19/803 versus 3% = 23/729, p = 0.353) (Table 1). Moreover, median gametocyte density by microscopy and *pfs25* transcripts showed no significant difference either (microscopy: 80 versus 460, p = 0.085; *pfs25*: 132.5 versus 210.9, p = 0.625) (Table 1).

**Table 1:** Prevalence and density of *P. falciparum* at baseline and endpoint

	Baseline	Endpoint	p value	OR/GMR (95%CI)
Parasite prevalence				
By microscopy	22/811 (3%)^a^	34/740 (5%)^b^	0.056	1.73 (0.97–3.13)
By PCR	49/811 (6%)	42/740 (6%)	0.829	0.94 (0.60–1.46)
By either test	52/811 (6%)	50/740 (7%)	0.838	1.06 (0.69–1.61)
Median parasitaemia/µL (IQR) (microscopy)	440 (80–1800)	860 (92–2690)	0.351	
Median 18S copies/µL (IQR) (qPCR) (n baseline = 33, endpoint = 29)	59.4 (7.8–1475.0)	713.5 (7.6–899.1)	0.095	
Gametocyte prevalence				
By microscopy	11/811 (1.4%)	15/740 (2.0%)	0.328	1.50 (0.64–3.65)
By *pfs25*	19/803 (2%)	23/729 (3%)	0.353	1.34 (0.69–2.63)
By either test	19/803 (2%)	23/729 (3%)	0.353	1.34 (0.69–2.63)
Gametocyte density (per μL)				
By microscopy (median) (IQR)	80 (48–280)	460 (80–860)	0.085	
By *pfs25* (geometric mean) (95%CI)	132.5 (30.2–580.5)	210.9 (56.8–782.7)	0.625	0.6 (0.1–4.2)

*OR* odds ratio, *GMR* geometric mean ratio

^a^Three and ^b^eight subjects were not confirmed by PCR during baseline and endpoint, respectively. This is presumably due to degraded DNA and possible LM false positive given 10 of 11 (90%) showed negative microscopy during cross check

Prevalence of *P. falciparum* gametocytes (*pfs25*) was significantly higher in microscopic than in the submicroscopic group (63% = 12/19 versus 28% = 7/25, p = 0.032, OR = 4.41, 95% CI 1.04–19.22). Moreover, the microscopic group demonstrated higher transcripts than submicroscopic, albeit statistically insignificant (geometric mean: 182.8, 95%CI 29.1–1150.5 versus 39.6, 95%CI 3.3–472.1, t-test, p = 0.39) (Fig. 3a).

#### Plasmodium vivax

Although microscopic examination showed no significant difference in the parasite prevalence between baseline and endpoint (5% = 43/811 versus 5% = 35/740, p = 0.643), PCR and either test demonstrated a significantly lower prevalence at the endpoint (PCR: 23% = 183/811 versus 18% = 135/740, p = 0.038; either test: 24% = 192/811 versus 19% = 142/740, p = 0.035) (Table 2). However, parasite density by both microscopy and qPCR did not show any significant difference between the two time points (median parasitaemia by microscopy: 112 versus 80, p = 0.832; median 18S gene copies: 192.2 versus 177.7, p = 0.719) (Table 2). No significant difference was seen in gametocyte prevalence by microscopy, *pvs25*, or either test (microscopy: 1.1% = 9/811 versus 1.9% = 14/740, p = 0.215; *pvs25*: 7% = 49/744 versus 5% = 39/704, p = 0.442, either test: 7% = 49/744 versus 5% = 39/704, p = 0.442) (Table 2). Moreover, gametocyte density by microscopy and *pvs25* transcripts did not demonstrate any significant difference (microscopy: 160 versus 64, p = 0.516, *pvs25* transcripts: 56.4 *versus* 65.6, p = 0.810) (Table 2).

**Table 2:** Prevalence and density of *P. vivax* at baseline and endpoint

	Baseline	Endpoint	p value	OR/GMR (95%CI)
Parasite prevalence				
By microscopy	43/811 (5%)^a^	35/740 (5%)^b^	0.643	0.89 (0.54–1.44)
By PCR	183/811 (23%)	135/740 (18%)	0.038*	0.77 (0.59–0.99)*
By either test	192/811 (24%)	142/740 (19%)	0.035*	0.77 (0.59–0.98)*
Median parasitaemia/µL (IQR) (microscopy)	112 (48–1000)	80 (16–1080)	0.832	
Median 18S copies/µL (IQR) (qPCR) (n baseline = 115, endpoint = 74)	192.2 (60.6–605.2)	177.7 (72.7–1535.0)	0.719	
Gametocyte prevalence				
By microscopy	9/811 (1.1%)	14/740 (1.9%)	0.215	1.72 (0.69–4.53)
By *pvs25*	49/744 (7%)	39/704 (5%)	0.442	0.83 (0.52–1.31)
By either test	49/744 (7%)	39/704 (5%)	0.442	0.83 (0.52–1.31)
Gametocyte density (per μL)				
By microscopy (median) (IQR)	160 (28–200)	64 (32–170)	0.516	
By *pvs25* (geometric mean) (95%CI)	56.4 (23.8–134.0)	65.6 (26.5–162.2)	0.810	0.9 (0.2–3.0)

*OR* odds ratio, *GMR* geometric mean ratio

^a^Nine and ^b^seven subjects were not confirmed by PCR during baseline and endpoint, respectively. This is presumably due to degraded DNA and possible LM false positive as 11 of 16 (69%) showed negative microscopy during cross check.

*p < 0.05

Gametocyte prevalence (*pvs25*) was significantly higher in microscopic than in submicroscopic group (92% = 23/25 versus 26% = 26/100, p < 0.001, OR = 32.73, 95% CI 7.07–296.20). Moreover, the microscopic group demonstrated higher transcripts than the submicroscopic group (geometric mean: 356.6, 95%CI 97.2–1309.8 versus 9.6, 95%CI 5.1–18.2, t-test, p < 0.001) (Fig. 3b).

### Dynamics of parasite and gametocyte at baseline and endpoint

Every parasitemic and gametocytemic subject was identified individually at each timepoint, and their infection status was followed or traced backwards. The treatment status was also linked for each identified individual.

#### Plasmodium falciparum

**Fig. 4:**
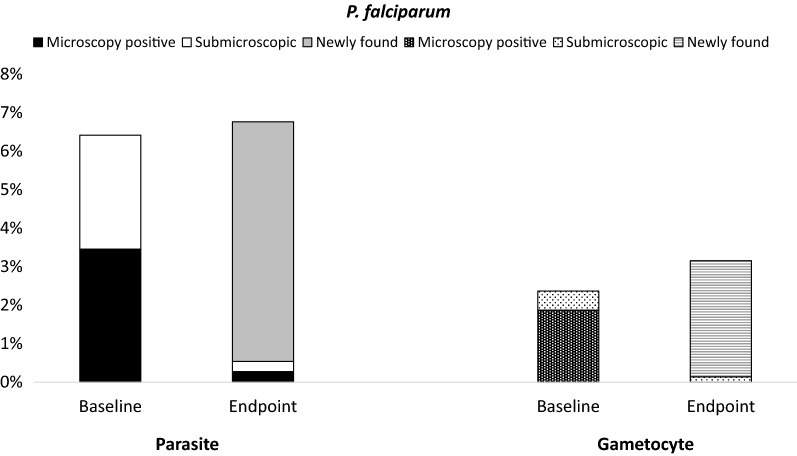
Dynamics of parasite and gametocyte in *P. falciparum* at baseline and endpoint. At baseline, *P. falciparum* prevalence was 6% (52/811) by microscopy and/or PCR. Half of these 52 subjects were positive by microscopy thus treated (54% = 28/52, ■), and 46% (24/52) were submicroscopic and untreated (□). At the endpoint, only two of each microscopic and submicroscopic group were positive. However, 46 subjects other than the mentioned groups were detected positive by microscopy (n = 32) or PCR (n = 14) ( ). Forty of these 46 subjects had data at baseline: 73% (n = 29) were parasite negative and 27% (n = 11) were infected with other species (*P. vivax*). Furthermore, similar pattern was observed in gametocyte. At baseline, gametocyte prevalence was 2% (19/803). Seventy-nine percent (15/19) had microscopic parasite and treated ( ), and 21% (4/19) had submicroscopic thus untreated ( ). At the endpoint, only one submicroscopic parasite remained positive. However, 22 subjects other than the mentioned groups were positive for gametocyte ( ). Twenty of these 22 subjects were negative at baseline while two had no data

Figure 4 demonstrates changes of parasite and gametocyte status of subjects between baseline and endpoint. Most subjects who were parasite or gametocyte positive at baseline were negative at the endpoint regardless of treatment. Conversely, most positive subjects detected during the endpoint were previously negative at baseline. Furthermore, some subjects showed different infecting species between the two time points, e.g. *P. vivax* followed by *P. falciparum* or vice versa. This demonstrates the dynamic nature of *Plasmodium* infection reservoirs in the area.

#### Plasmodium vivax

**Fig. 5:**
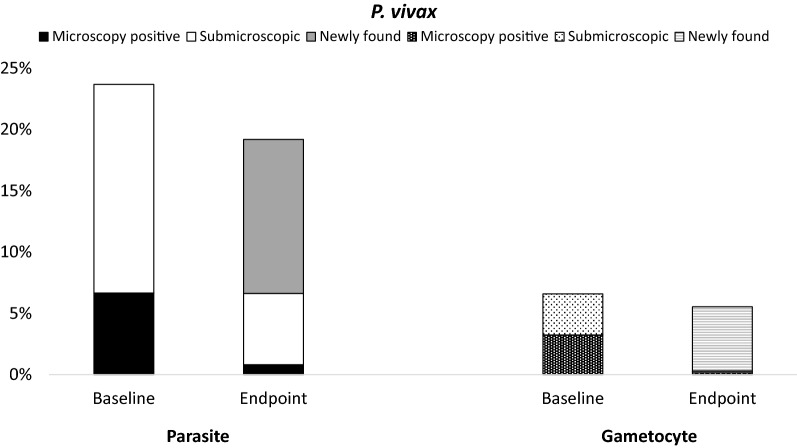
Dynamics of parasite and gametocyte in *P. vivax* at baseline and endpoint. At baseline, *P. vivax* prevalence was 24% (192/811) by microscopy and/or PCR. A third of these 192 subjects were microscopic positive thus treated (28% = 54/192, **■**) and 72% (138/192) were submicroscopic and untreated (□). At the endpoint, six of the microscopic and 43 submicroscopic subjects were positive. In addition, 93 positive subjects (23 by microscopy and 70 by PCR) other than those groups ( ) appeared. Eighty-five of these 93 subjects had data at baseline: 80 were parasite negative and 5 were infected with other species (*P. falciparum*). A similar pattern was also observed in gametocyte. At baseline, gametocyte prevalence was 7% (49/744). Fifty-three percent (26/49) had microscopic parasite and treated ( ), while 47% (23/49) had submicroscopic and untreated ( ). At the endpoint, only two (one microscopic and one submicroscopic) were positive. However, 37 subjects other than the mentioned groups were positive for gametocyte ( ). Thirty of these 37 subjects were negative at baseline, while seven had no data

Changes of parasite and gametocyte status between baseline and endpoint among the study subjects were also observed in *P. vivax* (Fig. 5). A large proportion of subjects who were parasite/gametocyte positive at baseline were negative during the endpoint regardless of treatment. Similarly, the majority of positive subjects identified at the endpoint were negative at baseline. Moreover, a change in infection species between the two time points was also demonstrated in some subjects, e.g. *P. vivax* followed by *P. falciparum* or vice versa.

## Discussion

This study investigated pre and post prevalence of parasite and gametocyte reservoirs of *P. falciparum* and *P. vivax* during 3-months of MST activities in East Nusa Tenggara, Indonesia. Dynamic temporal changes in species-specific infection among subjects occurred, but overall community-wide prevalence of parasites and gametocytes did not change. This indicates that the MST intervention with high compliance, though successful in treating diagnosed cases during its duration, was unsuccessful in reducing the parasite reservoir in the community to an extent that impacted overall transmission in this setting.

Complex infection dynamics surely influenced these findings. Studies have demonstrated that the numbers of circulating blood-stage parasites and gametocytes may fluctuate [6, 7] presenting the possibility that a proportion of infected subjects may have escaped a positive diagnosis with transient parasite levels below the detection limit at the point of blood collection (baseline/endpoint). Moreover, negativity in peripheral blood may conceal positivity in other compartments of the host (sequestration) that may later be released to peripheral blood [34] with vasculature of skin representing one possible such compartment [35, 36]. In the specific instance of *P. vivax,* there may be a considerable proportion of the population that are negative for parasitaemia but have dormant infections in their livers, i.e. latency [37]. New infections from the local carriers or introduced by travel from areas outside of the MST intervention area may also partly explain the findings. Natural clearing of parasites may have occurred among some of those who were positive at MST baseline (by PCR but not microscopy, i.e. not treated in the intervention) but negative at endpoint. The sum of these factors, of unknown proportional individual impacts, likely account for the apparently poor overall impacts on asexual and sexual parasitaemia prevalence rates resulting from the MST intervention.

In this context, a safe and efficacious MDA interventions of good coverage may be more effective than MST. Previous MDA studies have demonstrated that this approach may reduce incidence in low endemic malaria areas [23, 38]. Furthermore, the combination of MDA with vector control was shown to have a bigger impact in lowering incidence rates [23]. In addition, the prophylactic effect of the administered blood-schizonticide may prevent new infections for a certain period [19–21].

The primary limitation of this study is the limited sample size that reduces the power of the study. Furthermore, for a longitudinal study, the presence of more than two sampling time points would be preferable and better able to capture the temporal dynamics of the parasite reservoir in the study area.

This study demonstrates that there is a need to reconsider MST as a primary surveillance strategy, while alternative strategies such as MDA and complementary vector interventions should be considered and evaluated for efficacy in the drive towards malaria elimination.

## Conclusion

This study demonstrates the dynamic persistence of *P. falciparum* and *P. vivax* infectious reservoirs over the course of the MST intervention in West Timor, Indonesia. Overall, ongoing and continuing transmission is unaffected by the intervention. MDA with or without vector control may be considered as an alternative epidemiological intervention towards a more efficacious transmission reduction strategy.

## Supplementary Information

**Additional file 1.** Primer sequences and reaction conditions for 18S qPCR. The amplification and quantification of Pf- or Pv-specific 18S rRNA gene copies were conducted using SYBR-Green-based qPCR.

**Additional file 2.** Amplification and melting curve of gametocyte assay. The amplification and melting curve was generated by the 7500 Fast Real-Time PCR (Applied Biosystem) software.

**Additional file 3.** Performance of 18S qPCR and *pfs25*/*pvs25* RT-qPCR. The amplification and melting curve was generated by the 7500 Fast Real-Time PCR (Applied Biosystem) software. Mean CT, CT standard deviation, PCR efficiency, and r^2^ were calculated by running serial dilutions of plasmid in quintuples.

**Additional file 4.** Correlation between *pfs25*/*pvs25* transcript numbers and gametocyte density. A regression analysis was conducted to obtain the trendline for the relationship between transcript numbers and gametocyte density from samples with known gametocyte density.

## Data Availability

The dataset generated by this study is available from the corresponding author upon request.
