# Supplementary material for: Gametocyte carriage of Plasmodium falciparum (pfs25) and Plasmodium vivax (pvs25) during mass screening and treatment in West Timor, Indonesia: a longitudinal prospective study
Source: Malar J. 2021 Apr 9;20:177. doi: 10.1186/s12936-021-03709-y (PMC8034167; doi:10.1186/s12936-021-03709-y)
Supplement: Supplementary file 1 — Additional file 1. Primer sequences and reaction conditions for 18S qPCR. The amplification and quantification of Pf- or Pv-specific 18S rRNA gene copies were conducted using SYBR-Green-based qPCR. [file 12936_2021_3709_MOESM1_ESM.docx]

Additional File 1. RT qPCR and PCR methods

*pfs25*/*pvs25* RT qPCR

Transcriptor First Strand cDNA kit (Roche) was used to generate cDNAs for each sample in triplicates. During this first step, 4 µL RNA was mixed with 2 µL random hexamer, 1 µL anchored oligo (dT) primers, 4 µL RT buffer, 2 µL dNTP, 0.5 µL RNAse inhibitor, and 0.5 µL Reverse Transcriptase enzyme for a final volume of 20 µL. The incubation temperature was 10 min at 25^o^C, followed by 30 min at 55^o^C, then 5 min at 85^o^C. The tube was then immediately put on ice. The presence of mRNA transcript was verified using RT-qPCR targeting the 18S rRNA [1]. Only samples positive for 18S were analyzed for *pfs25* and *pvs25*. The *pfs25* and *pvs25* qPCR was conducted in 12 µL total volume containing 6 µL FastStart Essential DNA SYBR Green master (Roche), 4 µL cDNA, and 0.417 µM each of previously published primers [2]. The cycle conditions were as follows: 10 min at 95^o^C, followed by 45 cycles of 15 min at 95^o^C and 1 min at 58^o^C. T_M_ for *P. falciparum* was 74-75^o^C, and 79-80^o^C for *P. vivax*. For quantification, series of plasmid harboring target sequence with concentration of 10^5^, 10^4^, 10^3^, 10^2^, 10, 5, and 1 copy per reaction were run in triplicate and a standard curve generated for each run. Negative (no template) controls were included in triplicates.

18S rRNA qPCR

DNA were available in 77 of 83 *P. falciparum* and 202 of 231 *P. vivax* selected samples. These samples underwent SYBR Green-based qPCR targeting Pf- and Pv- specific 18S ribosomal unit gene. Primer sequences for *P. falciparum* were: 5’-TAT TGC TTT TGA GAG GTT TTG TTA CTT TG-3’ for forward, and 5’- ACC TCT GAC ATC TGA ATA CGA ATG C-3’ for reverse primer. As for *P. vivax*, the primer sequences were: 5’- GCT TTG TAA TTG GAA TGA TGG GAA T-3’ for forward primer, and 5’- ATG CGC ACA AAG TCG ATA CGA AG-3’ for reverse primer [3]. Reaction was performed on 7500 Fast Real-Time PCR system (Applied Biosystem) with 12 uL mix containing 1x SensiFast (Bioline UK) with 0.1 uM of each primer and 20 ng of DNA template for *P. falciparum*. For *P. vivax*, in a similar reaction volume and PCR buffer, 0.4 uM of each primer and 40 ng of DNA template was used. Serial dilution of plasmids harbouring target sequences with concentration of 10^5^, 10^4^, 10^3^, 10^2^, 10, 5, and 1 copy (copies) per reaction were run in duplicates as positive control as well as generating standard curve in each experiment. The conditions were 95^o^C for 10 minutes, followed by 45 cycles of denaturation at 95^o^C for 10 seconds, annealing at 64^o^C (Pf) or 58^o^C (Pv) for 30 seconds, and melting analysis by the software of 7500 Fast Real-Time PCR (Applied Biosystem). T_M_ value for *P. falciparum* was 77^o^C (+1^o^C), whereas Tm value for *P. vivax* was 81^o^C (+1^o^C). Quantity of the parasite was reported as number of DNA copies/µL. DNA were successfully amplified in 62 of 77 *P. falciparum* and 189 of 202 *P. vivax*.

References

1. Mangold KA, Manson RU, Koay ES, Stephens L, Regner M, Thomson RB, Jr., Peterson LR, Kaul KL: Real-time PCR for detection and identification of *Plasmodium spp*. J Clin Microbiol. 2005; 43**:**2435-2440.

2. Wampfler R, Mwingira F, Javati S, Robinson L, Betuela I, Siba P, Beck HP, Mueller I, Felger I: Strategies for detection of *Plasmodium* species gametocytes. PLoS One. 2013; 8**:**e76316.

3. Rosanas-Urgell A, Mueller D, Betuela I, Barnadas C, Iga J, Zimmerman PA, del Portillo HA, Siba P, Mueller I, Felger I: Comparison of diagnostic methods for the detection and quantification of the four sympatric *Plasmodium* species in field samples from Papua New Guinea. Malar J. 2010; 9**:**361.
