# Supplementary figures and images for: Gametocyte carriage of Plasmodium falciparum (pfs25) and Plasmodium vivax (pvs25) during mass screening and treatment in West Timor, Indonesia: a longitudinal prospective study
Source: Malar J. 2021 Apr 9;20:177. doi: 10.1186/s12936-021-03709-y (PMC8034167; doi:10.1186/s12936-021-03709-y)

Additional file 2. Amplification and melting curve of gametocyte assay

Pfs25


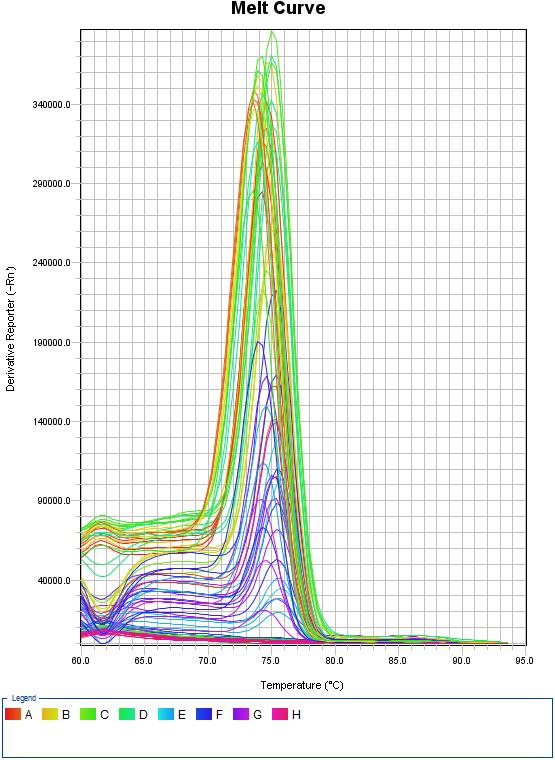


Pvs25


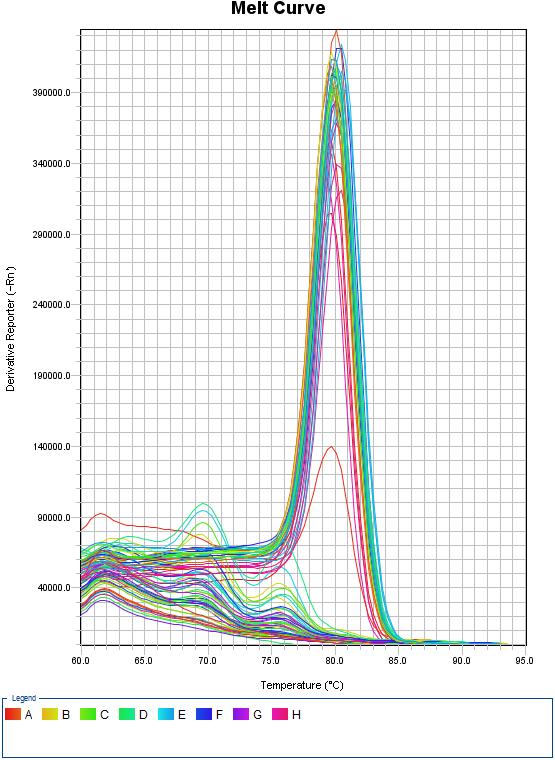

Supplement: Supplementary file 2 — Additional file 2. Amplification and melting curve of gametocyte assay. The amplification and melting curve was generated by the 7500 Fast Real-Time PCR (Applied Biosystem) software. [file 12936_2021_3709_MOESM2_ESM.docx]
