# Supplementary material for: Gametocyte carriage of Plasmodium falciparum (pfs25) and Plasmodium vivax (pvs25) during mass screening and treatment in West Timor, Indonesia: a longitudinal prospective study
Source: Malar J. 2021 Apr 9;20:177. doi: 10.1186/s12936-021-03709-y (PMC8034167; doi:10.1186/s12936-021-03709-y)
Supplement: Supplementary file 3 — Additional file 3. Performance of 18S qPCR and pfs25/pvs25 RT-qPCR. The amplification and melting curve was generated by the 7500 Fast Real-Time PCR (Applied Biosystem) software. Mean CT, CT standard deviation, PCR efficiency, and r2 were calculated by running serial dilutions of plasmid in quintuples. [file 12936_2021_3709_MOESM3_ESM.docx]

Additional file 3. Performance of RT-qPCR and 18S qPCR

The performance of qPCR is represented by the amplification efficiency (E) and coefficient of linearity (r^2^). Amplification efficiency is the number of target gene at the end of each PCR cycle divided by the number of that gene target at the start of cycle, expressed as the ratio to the maximum of such amplification, i.e. 2 or 100% (one strand will be amplified to 2 strands at each cycle). Coefficient of linearity is the coefficient of the standard curve linearity. Limit of detection (LOD) was measured by conducting experiment using plasmid control in quintuples. LOD is determined as the lowest plasmid copies/uL with minimum 3 of 5 plasmids were positive.

| Pfs25 | E= | 0,947 | r^2^= | 0,986 |  |  |  |
| --- | --- | --- | --- | --- | --- | --- | --- |
|  |  |  |  |  |  |  |  |
| Plasmid copies | 1. CT | 2. CT | 3.CT | 4.CT | 5.CT | Mean CT | SD |
| 100.000 | 20,93 | 20,81 | 20,72 | 20,81 | 20,47 | 20,748 | 0,15 |
| 10.000 | 24,73 | 24,65 | 24,41 | 24,42 | 24,32 | 24,506 | 0,16 |
| 1.000 | 28,7 | 28,49 | 28,45 | 28,43 | 28,43 | 28,5 | 0,10 |
| 100 | 30,92 | 30,65 | 30,95 | Neg | Neg | 30,84 | 0,13 |
| 10 | 35,75 | 33,19 | 33,5 | 35,37 | 35,74 | 34,14667 | 1,13 |
| 5 | 35,67 | Neg | Neg | 36,27 | Neg | - | - |
| 1 | Neg | Neg | Neg | Neg | Neg | - | - |

| Pvs25 | | E= | | 0,881 | | r^2^= | | 0,988 | |  | |  | |  |  |
| --- | --- | --- | --- | --- | --- | --- | --- | --- | --- | --- | --- | --- | --- | --- | --- |
| Plasmid copies | | 1. CT | | 2. CT | | 3.CT | | 4.CT | | 5.CT | | Mean CT | | SD |  |
| 100.000 | | 20,2 | | 20,22 | | 20,31 | | 20,12 | | 20,11 | | 20,192 | | 0,07 |  |
| 10.000 | | 23,35 | | 23,43 | | 23,67 | | 23,49 | | 23,64 | | 23,516 | | 0,12 |  |
| 1.000 | | 27,92 | | 27,44 | | 28,15 | | 27,97 | | 27,75 | | 27,846 | | 0,24 |  |
| 100 | | 32,39 | | 31,68 | | 31,92 | | 32,3 | | Neg | | 31,99667 | | 0,29 |  |
| 10 | | Neg | | 35,09 | | 34,65 | | 33,84 | | Neg | | 34,87 | | 0,52 |  |
| 5 | | Neg | | Neg | | Neg | | Neg | | Neg | | - | | - |  |
| 1 | | 37,06 | | Neg | | Neg | | Neg | | Neg | | - | | - |  |
| Pf18S | E= | | 0,877 | | r^2^= | | 0,983 | |  | |  | |  | | |
| Plasmid copies | 1. CT | | 2. CT | | 3.CT | | 4.CT | | 5.CT | | Mean CT | | SD | | |
| 100.000 | 20,93 | | 21,18 | | 20,94 | | 20,79 | | 20,44 | | 20,856 | | 0,24 | | |
| 10.000 | 24,9 | | 24,6 | | 24,23 | | 24,21 | | 24,09 | | 24,406 | | 0,30 | | |
| 1.000 | 27,96 | | 28,13 | | 27,64 | | 27,37 | | 27,46 | | 27,712 | | 0,29 | | |
| 100 | 32,36 | | 32,39 | | 32,3 | | 32,33 | | Neg | | 32,35 | | 0,03 | | |
| 10 | 35,68 | | 34,61 | | 35,71 | | Neg | | Neg | | 35,33333 | | 0,51 | | |
| 5 | 38,47 | | 38,2 | | Neg | | 36,13 | | Neg | | 37,6 | | 1,045275 | | |
| 1 | 36,66 | | Neg | | 38,6 | | Neg | | Neg | | - | | - | | |

| Pv18S | E= | 1,029 | r^2^= | 0,99 |  |  |  |
| --- | --- | --- | --- | --- | --- | --- | --- |
| Plasmid copies | 1. CT | 2. CT | 3.CT | 4.CT | 5.CT | Mean CT | SD |
| 100.000 | 17,57 | 17,67 | 17,66 | 17,59 | 17,51 | 17,6 | 0,06 |
| 10.000 | 21,11 | 20,88 | 20,98 | 21,1 | 21,07 | 21,028 | 0,09 |
| 1.000 | 24,62 | 24,68 | 24,71 | 24,37 | 24,65 | 24,606 | 0,12 |
| 100 | 28,4 | 28,93 | 28,49 | 28,16 | 28,23 | 28,60667 | 0,27 |
| 10 | 30,45 | 30,76 | 30,7 | 31 | 30,38 | 30,63667 | 0,22 |
| 5 | Neg | 31,46 | Neg | 32,6 | 31,23 | 31,76333 | 0,599018 |
| 1 | Neg | Neg | 32,8 | Neg | Neg | - | - |
