# Supplementary material for: Gametocyte carriage of Plasmodium falciparum (pfs25) and Plasmodium vivax (pvs25) during mass screening and treatment in West Timor, Indonesia: a longitudinal prospective study
Source: Malar J. 2021 Apr 9;20:177. doi: 10.1186/s12936-021-03709-y (PMC8034167; doi:10.1186/s12936-021-03709-y)
Supplement: Supplementary file 4 — Additional file 4. Correlation between pfs25/pvs25 transcript numbers and gametocyte density. A regression analysis was conducted to obtain the trendline for the relationship between transcript numbers and gametocyte density from samples with known gametocyte density. [file 12936_2021_3709_MOESM4_ESM.pptx]

## Slide 1
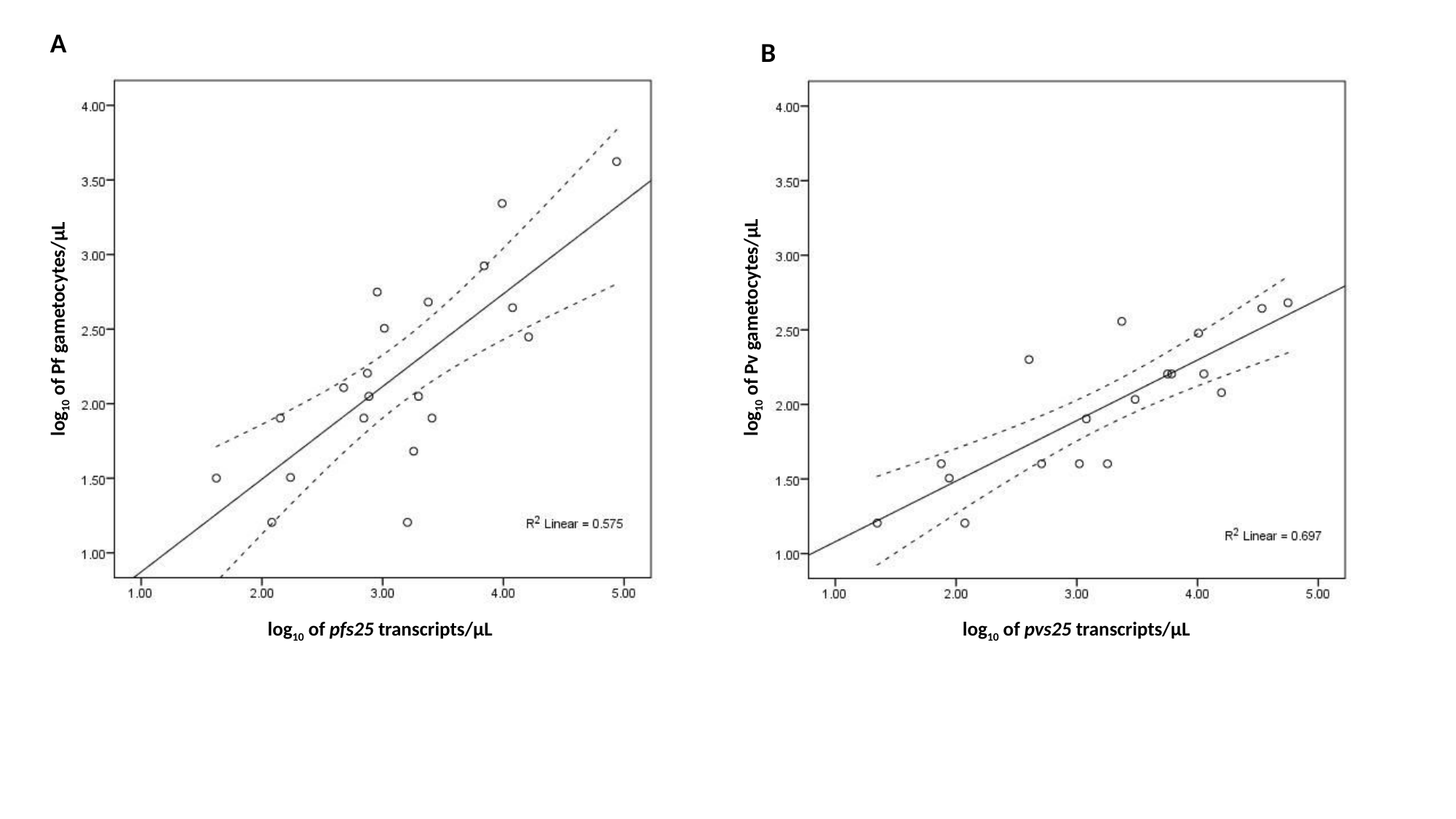

A
B
log10 of Pv gametocytes/µL
log10 of Pf gametocytes/µL
log10 of pfs25 transcripts/µL
log10 of pvs25 transcripts/µL
